# Evaluation of hypoxia in a feline model of head and neck cancer using ^64^Cu-ATSM positron emission tomography/computed tomography

**DOI:** 10.1186/1471-2407-13-218

**Published:** 2013-04-30

**Authors:** Elizabeth A Ballegeer, Nicole J Madrill, Kevin L Berger, Dalen W Agnew, Elizabeth A McNiel

**Affiliations:** 1 Department of Small Animal Clinical Sciences, Michigan State University, East Lansing, MI, 48824, USA; 2 Department of Pathobiology and Diagnostic Investigation, Michigan State University, East Lansing, MI, 48824, USA; 3 Chesapeake Medical Imaging, Annapolis, MD, 21401, USA; 4 Tufts Cummings School of Veterinary Medicine and Molecular Oncology Research Institute, Boston, MA, 02111, USA

**Keywords:** Hypoxia, Head and neck cancer, Feline, ^64^Cu-ATSM PET/CT, O_2_ probe, Pimonidazole

## Abstract

**Background:**

Human and feline head and neck squamous cell carcinoma (HNSCC) share histology, certain molecular features, as well as locally aggressive and highly recurrent clinical behavior. In human HNSCC, the presence of significant hypoxia within these tumors is considered an important factor in the development of a more aggressive phenotype and poor response to therapy. We hypothesized that feline head and neck tumors, particularly HNSCC, would exhibit hypoxia and that ^64^Cu-diacetyl-bis(N4-methylthiosemicarbazone) (Cu-ATSM) positron emission tomography/computed tomography (PET/CT) would permit detection of intratumoral hypoxia.

**Methods:**

12 cats with measureable head and neck tumors were given ^64^Cu-ATSM and iodinated contrast for PET/CT scan. The presence or absence of hypoxia was also assessed using an intratumoral fluorescent life-time probe to quantitate pO_2_ and pimonidazole immunohistochemical staining in biopsy specimens. In two cats, intratumoral O_2_ and ^64^Cu-ATSM uptake was measured before and after treatment with anti-angiogenic agents to determine the effect of these agents on hypoxia.

**Results:**

Eleven of twelve feline tumors demonstrated significant ^64^Cu-ATSM uptake, regardless of malignant or benign etiology. The presence (and absence) of hypoxia was confirmed using the fluorescent O_2_ detection probe in nine tumors, and using pimonidazole staining in three tumors. Squamous cell carcinomas (HNSCC) demonstrated the highest degree of hypoxia, with T_max_/M ratios ranging from 4.3 to 21.8. Additional non-neoplastic tissues exhibited ^64^Cu-ATSM uptake suggestive of hypoxia including reactive draining lymph nodes, non-malignant thyroid pathology, a tooth root abscess, and otitis media. In two cats with HNSCC that received anti-vascular agents, the pattern of ^64^Cu-ATSM uptake was altered after treatment, demonstrating the potential of the feline model to study the modulation of tumor oxygenation.

**Conclusion:**

Feline HNSCC serves as a clinically relevant model for the investigation of intratumoral hypoxia including its measurement, modulation and targeting.

## Background

Hypoxia occurs in tumors for a variety of reasons; these include abnormal vessel growth [1,2], fluid accumulation in the tumor extracellular matrix and rapid proliferation of cancer cells causing high interstitial pressure [2,3], a breakdown of the diffusion geometry within the tumor, and paraneoplastic or therapy-related anemia leading to decreased oxygen delivery [4]. While tumor hypoxia was initially recognized as a cause for cellular radiation resistance, it is now known to contribute more generally to malignant progression and therapeutic failures [5-7]. Lack of oxygen within tumors results in relative resistance to ionizing radiation, since the presence of oxygen permits irreversible peroxidation of DNA following ionizing radiation [5]. Furthermore, in acidic, hypoxic conditions, an aggressive cellular phenotype, with increased propensity for angiogenesis, invasion, and metastasis can emerge, an effect mediated by hypoxia-inducible transcription factors [2,8-11].

Hypoxia and its contribution to malignant phenotype and treatment failure are well-documented in head and neck squamous cell carcinoma (HNSCC) [6,9,11-17]. Conversely, modulation of hypoxia may provide benefit to patients with HNSCC [18], which underscores the importance of understanding the impact of therapies on tumor hypoxia and developing improved methods to modulate tumor pO_2_ and the molecular response to hypoxia. Unfortunately, animal models used to study HNSCC may not completely recapitulate the larger, invasive, and metastatic phenotype observed in human clinical populations. Indeed for many cancers and agents, there is a significant gap between preclinical rodent investigations and the clinical response of patients, suggesting a need to understand the biology of therapeutic interventions in models that more closely mimic human malignancies.

One potential model for HNSCC is head and neck squamous cell carcinoma that occurs spontaneously in pet cats. HNSCC is among the most common cancers affecting cats [19,20]. Although its causation is not well studied, it is thought that the fastidious grooming behavior exhibited by cats may put the feline oropharynx at risk of exposure to a variety of environmental carcinogens [21-23]. In addition to sharing histopathologic appearance, feline HNSCC is characterized by invasive, highly recurrent, and sometimes metastatic phenotype that is also observed in people with this cancer [19]. Furthermore, feline and human HNSCC may share their molecular underpinnings including frequent expression of EGFR [24,25] and Cox-2 [26-28], as well as mutant p53 [23]. However, to our knowledge, the presence of hypoxia has not been previously studied in feline HNSCC.

A great variety of techniques to detect hypoxia in tumors have been developed. Traditionally, techniques for evaluating tumor hypoxia have comprised tissue probes and immunohistochemical evaluation of tissue [29]. However, these methods have limited clinical application given that they are invasive and provide only focal assessment of oxygenation. To provide a clinically applicable, global assessment of tumor hypoxia, imaging techniques have been applied. *In vivo* imaging methods include both magnetic resonance (MR) techniques such as dynamic contrast enhanced-MR and nuclear-based imaging modalities, including SPECT (Single Photon Emission Computed Tomography) and PET (Positron Emission Tomography).

PET utilizes the detection of secondary, annihilation photons produced by cyclotron-generated, positron-emitting radionuclides, such as ^18^F, ^13^N, ^15^O, ^11^C, ^62^Cu, and ^64^Cu. Suitable radionuclides are chemically coupled with tracers targeted for detection of particular molecular or physiologic parameters, such as hypoxia. Though activity of the most commonly used PET agent, 2-deoxy-2-(^18^F)fluoro-D-glucose (FDG), has been correlated with gene expression induced by hypoxia (HIF-1 α), FDG does not directly detect hypoxia within the tissues [17]. A number of PET tracers specifically designed for the detection of hypoxia have been developed. These include either misonidazole (MISO) or azomycinarabinofuranoside (AZA) coupled to ^18^F, or ATSM coupled to a positron-emitting isotope of Cu (^62^Cu of ^64^Cu) [13-16,30,31]. All such agents rely on the hypoxia-dependent trapping of the tracer in cells that are hypoxic, yet viable. Cu-diacetyl-bis(N_4_-methylthiosemicarbazone) (Cu-ATSM) has been demonstrated to exhibit hypoxia associated cellular uptake and is particularly advantageous due to its rapid uptake and strong signal to noise ratio. However, there is also evidence that some tumor subtypes may not demonstrate a direct relationship between Cu-ATSM signal and hypoxia [16,32].

Our primary goal was to determine whether feline head and neck tumors, particularly feline HNSCC, exhibit biologically relevant hypoxia. For our purposes we considered levels of hypoxia sufficient to confer cellular radioresistance or to induce of HIF1α signaling to be biologically relevant. Such consequences occur below 1% O_2_ (7.5 mmHg). In addition, we planned to evaluate the utility of ^64^Cu-ATSM PET to detect hypoxic tumors in cats. To accomplish these aims, all cats were imaged with ^64^Cu-ATSM PET/CT and were also evaluated using at least one other technique to measure intratumoral hypoxia including a fluorescent probe and/or immunohistochemical detection of pimonidazole. Herein, we demonstrate that most feline head and neck tumors concentrate ^64^Cu-ATSM and that this signal is concomitant with low intratumoral oxygen levels and pimonidazole uptake. Feline HNSCC provides an opportunity to explore the modulation of tumor oxygen and vascular physiology in a clinically relevant system.

## Methods

### Animals

This study was conducted with approval from Michigan State University’s Institutional Animal Care and Use Committee and informed client consent. Twelve pet cats with head and neck tumors were recruited for participation in this study. Inclusion criteria were the presence of a measureable and accessible tumor and lack of systemic illness that would preclude anesthesia or would impact oxygenation (e.g. severe anemia, respiratory disease). Initial evaluation included a physical examination, complete blood count, serum biochemical profile, and urinalysis.

### Anesthesia

Cats were anesthetized for PET/CT and then the following day for intratumoral oxygen probe measurements and tumor biopsy. In order to allow cats to breathe room air and not 100% oxygen, injectable rather than gas anesthesia was used for PET and intratumoral O_2_ measurements. Cats were switched to either Isoflurane (1–3% in oxygen) or desflurane (5–9% in oxygen) anesthesia immediately prior to biopsy. Cats were placed under general anesthesia using either a combination of diazepam (0.5 mg/kg)/ketamine (10 mg/kg) or a continuous rate propofol infusion (100 – 600 μg/kg/min to effect). Decisions regarding anesthetic combination were made based on the physical status and concurrent conditions of these older, in many cases geriatric, cats. Diazepam/ketamine combinations were augmented with either butorphanol (0.2 mg/kg), buprenorphine and or dexmedetomidine (40 μg/kg) for improved immobilization. Cats were continuously monitored visually and for heart rate, respiratory rate, and oxygenation via a pulse-oximeter. Cats that received dexmedetomidine were given atipamezole (250 μg/kg) intramuscularly for reversal of sedation upon completion of the procedure.

### PET/CT

^64^Cu-ATSM was produced with a commercially available ligand kit (Proportional Technologies, Houston, TX) using manufacturer instructions and 64-Cu obtained from the Washington University Medical Center cyclotron. The target dose was 74 MBq (2 mCi) of ^64^Cu- ATSM per cat with actual dose ranging from 72.5 to 107 MBq (1.96 to 2.9 mCi) delivered intravenously through a catheter placed in either the cephalic or saphenous vein. Scans were performed following an uptake period of 20 minutes. Following induction of general anesthesia, cats were positioned in sternal recumbency in a GE Discovery™ STE PET/CT scanner (GE Healthcare). After a CT attenuation correction scan was performed, PET imaging of the head and thorax were performed in two, 15.7 cm bed positions, with 3D acquisition parameters. Intravenous non-ionic iodinated contrast (iohexol) was administered at a dosage of 660 mg I/kg for a post-emmission CT scan.

### Intratumoral oxygen measurement

To quantify pO_2_ in particular locations within the tumor, a fluorescent life-time probe (OxyLab pO_2_™, Oxford Optronix, Oxford, England, UK) was used in conjunction with a large area needle sensor to provide pO_2_ sampling area of 0.8 – 1.0 mm^2^. PO_2_ was measured at three distinct regions within each tumor. To perform the measurement, a 22-gauge over- the-needle intravenous catheter was used as a guide for the O_2_ sensor. The catheter was introduced into the tumor and the catheter needle was retracted, leaving the polypropylene sheath in place. The 23-gauge sensor was passed through the catheter to embed within the tumor parenchyma beyond the catheter opening. The probe was left in place until pO_2_ readout stabilized, with less than 1–2 mmHg variation for a two minute period. Several minutes were required to equilibrate at each location. The value reached at the equilibration point was recorded as the pO_2_ for that region. This process was repeated to obtain three pO_2_ measurements at distinct locations. In two instances, only two measurements were obtained due to the small volume of accessible tumor. Location of the probe was documented in the cases treated with antiangiogenic agents and reevaluated, using a diagrammatic representation of the feline oral cavity and using digital photography to reproduce the area probed as accurately as possible.

### Pimonidazole immunohistochemistry

There are no published feline doses for pimonidazole. Therefore dose was based on that reported in the dog [33,34]. Pimonidazole was administered intravenously at the time of ^64^Cu-ATSM administration (24-hours before biopsy) at a dose of 0.28 mg/m^2^ and 0.5 mg/m^2^ in four and five cats, respectively. In three cats, pimonidazole was administered at a dose of 0.5 mg/m^2^ IV between 20 and 60 minutes prior to biopsy. Biopsies were collected 24 hours following the PET/CT imaging and immediately following pO_2_ probe measurements. Following collection, biopsies were fixed in 4% paraformaldehyde at 4°C for 24 hours. Samples were then transferred to distilled water, 30% ethanol, 50% ethanol and 70% ethanol in series, each for 24 hours at 4°C. The fixed specimens were embedded in paraffin, sectioned onto slides, and stained using a commercially available monocolonal antibody against pimonidazole tissue adducts ((Hypoxyprobe™- 1, Hypoxyprobe Inc, Burlington, MA) according to manufacturer instructions. Simultaneous examination of H&E stained sections was performed using light microscopy by a board-certified veterinary pathologist (DWA). Samples were scored to determine the proportion of tumor cells exhibiting pimonidazole binding, as previously described [35].

### Vascular targeting

Two cats were treated with vascular targeting agents and evaluated with ^64^Cu-ATSM PET/CT before and after treatment. Pre- and Post- treatment imaging was performed 7 days apart. The first agent evaluated was an antivascular peptide, Anginex, that targets galectin-1 on the surface of endothelial cells [36]. Anginex was administered subcutaneously at a dose of 5 mg/kg twice daily for a total of 5 doses prior to the second scan. The second agent used was a multiple tyrosine kinase inhibitor, toceranib (Palladia®, Pfizer Animal Health, Kalamazoo, MI) that targets vascular endothelial growth factor receptor 2 (VEGFR2) as well as platelet-derived growth factor 2 and c-KIT. Toceranib was administered at a dose of 2.7 mg/kg per os, every other day for a total of three treatments prior to repeating the PET/CT.

### Imaging data analysis

PET/CT data was analyzed with MedImage Medview™ LE version 11.7, by a board-certified veterinary radiologist (EAB). Regions of interest were hand-drawn around each tumor and within dorsal cervical muscles, to determine maximum and average tumor uptake (T_max_ and T_av_), and average muscle uptake (M). These are standardized uptake values (SUV_bw_) normalized for body weight; SUV is the is the ratio of the decay corrected activity per unit volume of tissue (nCi/ml) to the administered activity per unit of body weight (nCi/g) [37]. Ratios of uptake of tumor to uptake of muscle were calculated (T_max_/M and T_av_/M) as relative measures of tumor hypoxia.

### Statistical analysis

All numerical variables were tested for deviation from a normal distribution using the D’Agostino Pearson Test. Data were described using a median value or using mean ± standard deviation, if they failed or passed normality testing, respectively. The Mann-Whitney test was used to compare T_max_/M and T_av_/M between HNSCC and other tumor types. A Kruskal Wallis test was used to compare T_max_/M and T_av_/M in between HNSCC, sarcomas and benign tumors.

## Results

The twelve cats included in this study ranged in age from 7–16 years (mean = 12 ± 2.8 years), comprised 8 females and 4 males, and were all of common domestic (rather than purebred) origin. Of the twelve primary masses examined in the cats, six were squamous cell carcinomas (HNSCC), three were sarcomas, and three were benign lesions, (Table 1). Size of the masses ranged from 1.4 cm (benign) to 8.7 cm (malignant) maximum diameter with a mean of 4.0 ± 2.0 cm.

**Figure 1:**
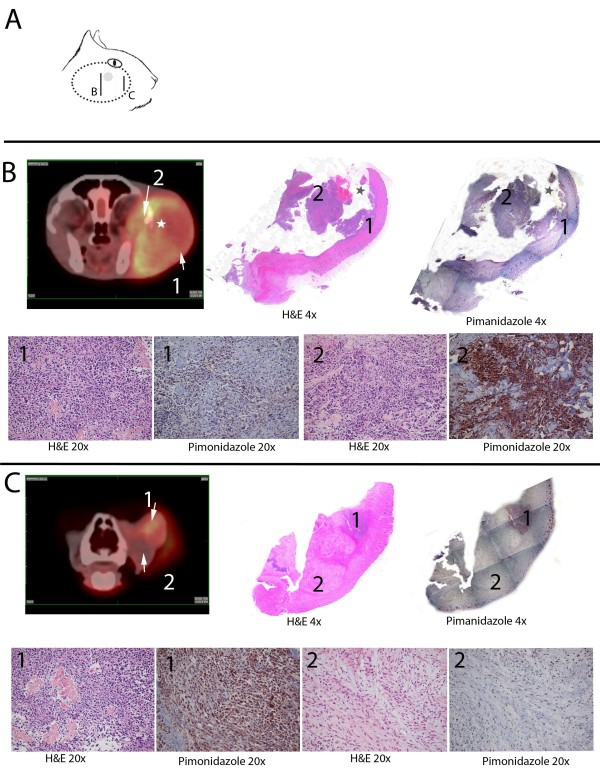
**Spatial Correlation between **^**64**^**Cu-ATSM and pimonidazole uptake in a cat with maxillary ostesarcoma.** Formalin-fixation and sectioning of the entire tumor from cat #12 was performed to compare spatial distribution of pimonidazole in relation to ^64^Cu-ATSM uptake on PET. Panel **A**: Diagrammatic representation of a 5.1-cm osteosarcoma on the right lateral maxilla of a 7 year old spayed female domestic shorthair cat. The position of two transverse sections are indicated by the letters B and C are shown in the diagram. The imaging and histologic sections at these locations are provided in the panels below. Panels **B** and **C**: Top row: Transverse fused PET/CT image (left). H&E stained tissue section at 4× magnification (middle). Pimonidazole at 4× magnification (reconstructed from tiled images) stained tissue section (right). Corresponding regions in the PET/CT and histologic sections are marked by the numbers 1 and 2. Bottom Row (20× magnification of histologic sections): H&E stained image from area marked “1” (Far left); Pimonidazole stained image from area marked “1” (Middle left). H&E stained image from area marked “2” (Middle right); Pimonidazole stained image from area marked “2” (Far right). Note: The tumor tissue was friable and there were areas of necrotic debris, such as the area marked by a star in panel **B**, that were lost during processing.

With the exception of the bone cyst, all lesions demonstrated at least regional 64-Cu uptake (Table 1). Tmax/M ratios were significantly higher (P < 0.005) than Tav/M ratios, reflecting heterogeneity of uptake in tumors, which in three tumors (both osteosarcomas and one HNSCC) included signal voids. For the tumors exhibiting signal voids, pre and post contrast CT images were compared. Based on Hounsfield Unit (HU) analysis, the tumoral regions exhibiting no 64-Cu uptake were also devoid of CT contrast enhancement, which demonstrates lack of perfusion and, likely, necrosis. Pre and post contrast measurements in the HNSCC were 41 and 40 HU respectively, while in the osteosarcoma, in an area without mineral attenuation, values were 40 and 42 HU pre and post contrast; this compares to an area with contrast enhancement and 64-Cu uptake in the same tumor, of 37 and 122 HU pre and post contrast. In the second osteosarcoma, histopathology of the entire tumor was performed (Figure 1) and this demonstrated that the signal void occurred within a necrotic cavity communicating with a cutaneous ulcer.

**Table 1:** Measurement of tumor hypoxia in twelve feline head and neck tumors

**Cat:**	**Diagnosis**	**Location**	**Maximum dimension (cm)**	**Tmax/M**	**Tav/M**	**% PIM**	**pO2 1 (mmHg)**	**pO2 2 (mmHg)**	**pO2 3 (mmHg)**
**1**	Polyp	Mandible	1.93	6.0	1.9	NE	32	5.5	0.6
**2**	Bone cyst	Maxilla	1.46	1.4	1.0	NE	61	68	NE
**3**	Eosinophilic granuloma	Sublingual	1.37	6.4	3.0	NE	NE	NE	NE
**4**	SCC	Maxilla	4.16	14	4.7	NE	1.7	4.73	NE
**5**	SCC	Mandible	4.32	11	4.8	50%	NE	NE	NE
**6**	SCC	sublingual	3.37	4.8	2.2	60%	1.8	40	0.8
**7**	SCC	Maxilla	4.66	22	5.2	NE	50	0.3	3.3
**8**	SCC	Mandible	4.41	11	3	NE	2.2	26.3	2.6
**9**	SCC	Maxilla	4.18	4.3	1.8	NE	0.3	0	0.5
**10**	FSA	Maxilla	4.42	7.3	3.3	NE	0.4	0.8	0.38
**11**	OSA	Maxilla	8.73	7.5	1.5	NE	6.5	10.7	2.1
**12**	OSA	Maxilla	5.11	6.2	2.2	Fig 1	NE	NE	NE

Cats were assigned an arbitrary number from 1–12. The underlying etiology of the mass, location of the mass, maximum dimension of the mass, as well as values for the three diagnostic tests are provided. Tmax/M is a ratio of maximum 64Cu-ATSM uptake over muscular uptake as a normalization for signal to background uptake, Tav/M is the average uptake over the entire mass, %PIM is the percentage of pimonidazole uptake, and pO2 is the measured oxygen pressure with a fluorescent life-time probe. *HNSCC* = squamous cell carcinoma, *FSA* = fibrosarcoma, *OSA* = osteosarcoma, *NE* = not evaluated, due to technical error.

^64^Cu - ATSM uptake was highest for HNSCC (Median T_max_/M = 11; Median T_av_/M = 3.8) than for sarcomas (Median T_max_/M = 7.3; Median T_av_/M = 2.2) and the benign masses (Median T_max_/M = 6.0; Median T_av_/M = 1.9). However, given the small numbers and variability in the data, there were no statistically significant differences in comparing uptake parameters between HNSCC (P = 0.24 for T_max_/M; P = 0.09 for T_av_/M) and other tumor types or between malignant and benign tumors (P = 0.15 for T_max_/M; P = 0.21 to T_av_/M).

Quantitative detection of tumor O_2_ using the intratumoral fluorescent probe confirmed, using a different technique, that tumors with ^64^Cu-ATSM uptake also exhibit regions of very low oxygenation, ranging from 0.6 to 2.6 mmHg, which would be expected to have biologic consequences including radioresistance and HIF1α induction (Table 1). Conversely, the tissues in the region of the bone cyst that did not take up ^64^Cu-ATSM, appeared to be normoxic (Table 1).

In addition to the fluorescent O_2_ detection probe, pimonidazole immunohistochemistry was also used to investigate tumor hypoxia. When pimonidazole was administered 24 hours prior to biopsy, there was minimal detectable immunostaining in samples, regardless of dose. Whereas in three tumors, in which pimonidazole was administered within an hour of biopsy, there was intense immunohistochemical staining. The discrepancy in staining between samples collected 24 hours or 1 hour before biopsy suggests that pimonidazole tissue adducts are relatively short-lived in cats [33]. The patient with osteosarcoma was severely compromised by the primary tumor and systemic metastasis and died following imaging. Thus the entire tumor was available for examination and spatial comparison of pimonidazole and ^64^Cu-ATSM uptake (Figure 1). This comparison suggests a similar distribution of pimonidazole and ^64^Cu-ATSM in this tumor.

Several additional tissues, distinct from the primary tumor, demonstrated ^64^Cu-ATSM uptake, including lymph nodes (medial and lateral retropharyngeal lymph nodes, mandibular lymph nodes, and superficial cervical lymph nodes) draining the primary tumor in six of the cats with malignancies. In one of these six cats, there was additional assessment of a mandibular lymph node evaluated by fine needle aspiration cytology, which demonstrated reactive change rather than metastatic neoplasia.

Two of the cats had fluid within the tympanic bulla that demonstrated ^64^Cu-ATSM uptake. One cat demonstrated signal associated with a necrotic maxillary molar. Three of the cats had ^64^Cu-ATSM uptake within the thyroid glands. In one cat with bilateral thyroid uptake, clinical hyperthyroidism was confirmed by serum thyroid panel. In another case, a large thyroid gland with increased ^64^Cu-ATSM uptake on PET/CT was confirmed as a thyroid adenoma at necropsy. In the third cat, there was PET signal in an enlarged thyroid gland, but disease was not confirmed with serum panel or histopathology. The cat with osteosarcoma that died immediately following PET/CT had a diffuse increase in pulmonary signal and at necropsy there were multiple 2–4 mm metastatic nodules in its lungs.

**Figure 2:**
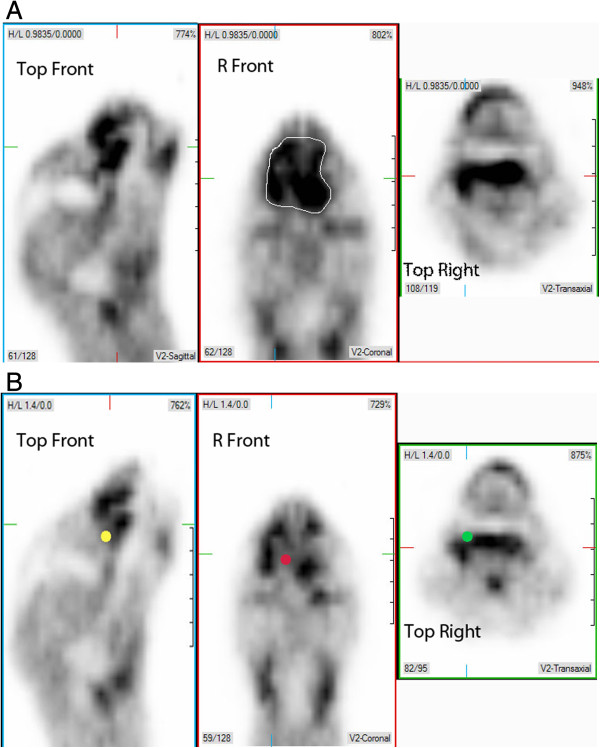
**Uptake of **^**64**^**Cu-ATSM within a maxillary squamous cell carcinoma.** PET signalis presented in three planes of imaging; sagittal plane image on the left, dorsal plane image in the middle, and transverse plane on the right. A similar area of transection through the head in each plane was chosen between two time points, using anatomic landmarks of the orbit, mandibular rami, and medial canthus of the palpebrae. 2A represents the mass before treatment with toceranib, 2**B** 7 days post treatment. In 2**A**, the mass is best seen as a large area of ATSM uptake on dorsal plane PET image (white outline). Note the region of decreased uptake within the ventromedial portion of the mass, represented by the red dot on dorsal plane PET image, yellow dot on sagittal plane PET image, and green dot on transverse plane PET image.

In two cats, intratumoral hypoxia was evaluated before and after treatment with an antiangiogenic agent, either a galectin-1 targeted peptide (Anginex) or a multiple tyrosine kinase inhibitor that targets VEFGR2 (toceranib, Palladia™, Pfizer Animal Health, Kalamazoo, MI). PET/CT and intratumoral oxygen probe measurements were performed one week apart with treatment administered in the intervening interval. Similar location of the probe was attempted as outlined in the materials and methods. After one week, there was minimal change in tumor size as measured by CT, with both tumors classifiable as “stable” when applying the RECIST (Response Evaluation Criteria in Solid Tumors) system used for human tumors [38]. Nor was there appreciable change in CT appearance. However, both tumors exhibited a slight increase in T_max_/M. While T_av_/M increased slightly in the Anginex-treated cat, there was a slight decrease in T_av_/M in the toceranib-treated cat, with select regions of this second tumor exhibiting less radiopharmaceutical uptake (see Figure 2; Table 2). Intratumoral probe measurements demonstrated variability in certain regions of both tumors (Table 2). In the toceranib-treated tumor, pO_2_ values were consistently increased at each location. In the Anginex- treated tumor the three regional measurements demonstrated decreased, increased, and stable pO_2_ levels, respectively.

**Table 2:** Evaluation of hypoxia in feline SCC before and after anti-angiogenic therapy

**Column1**	**Diagnosis**	**Location**	**Maximum dimension (cm)**	**Tmax/M**	**Tav/M**	**pO2 #1 (mmHg)**	**pO2 #2 (mmHg)**	**PO2 #3 (mmHg)**
**Cat 8a**	SCC	Mandible	4.41	**11**	3.05	2.2	26	2.6
**Cat 8b**	SCC	Mandible	4.41	**11.8**	3.16	24	2.8	2.6
**Cat 9a**	SCC	Maxilla	4.18	**4.25**	1.83	0.3	0.1	0.6
**Cat 9b**	SCC	Maxilla	4.06	**5**	1.73	14	19	20

Cat 8 was treated with Anginex, an anti-vascular peptide, while cat 9 was treated with toceranib, a VEGFR2 inhibitor. 64Cu-ATSM PET/CT and intratumoural fluorescent O2 measurements were performed 7 days apart, with treatment occurring in the intervening interval. Lower case letter a and b indicates pre- and post-treatment data, respectively. The location of the mass, maximum dimension of the mass, Tmax/M, Tav/M and pO2 in three tumor regions are provided. *HNSCC* = head and neck squamous cell carcinoma.

## Discussion

The biologic effects and clinical consequences of intratumoral hypoxia have been the focus of decades of research. It is well-established that hypoxic cells *in vitro* and in animals are relatively radiation resistant [39]. Furthermore, it has been demonstrated that patients with hypoxic tumors, including HNSCC, are more likely to experience treatment failures both locally and systemically [12,18,39]. Therefore, a variety of methods to increase tumor oxygenation or to target hypoxic cells within tumors have been investigated. Traditionally, these efforts have included measures such as hyperbaric oxygen administration, inhalation of carbogen gas, and the use of nitroimidazoles as hypoxic cell radiation sensitizers [18]. More recently, agents that specifically target hypoxic cell populations have been developed [40]. Finally, it has also been observed that anti-angiogenic and anti-vascular therapies may also modulate tumor oxygenation [1,41]. However, despite these various efforts, clinical gains have been modest. While a multitude of factors may contribute to the gap between experimental and clinical results, two issues are particularly problematic. First, of particular importance in the targeting of tumor hypoxia, the assessment of relevant molecular and biologic surrogate endpoints is challenging in humans [42]. Second, rodent models for human cancer have significant limitations that do not always permit direct clinical translation [43]. In this study, we demonstrate the application of developing technology to assess tumor oxygenation in a clinically relevant model, spontaneous feline HNSCC.

There are a variety of methods for evaluating tumor oxygenation and these have been thoroughly reviewed elsewhere [29,42]. All of these techniques have strengths and limitations, with no single technique offering complete characterization of this dynamic, complex phenomenon [42]. Imaging technology, by providing a noninvasive, three-dimensional, real-time assessment of hypoxia, is particularly promising as a clinical tool. In this study, we investigated hypoxia using ^64^Cu-ATSM. Cu(II)-conjugated ATSM enters cells by either passive diffusion or endocytosis where is reduced and trapped, likely with the dissociation of reduced Cu(I) from ATSM, within hypoxic, yet viable cells [44,45]. Normoxic cells are able to oxidize the reduced copper, which then is transported out of cells, either passively or, more likely, using a variety of chaperones or transporters [42,45]. In preclinical studies, data demonstrate that tumor cells vary in their uptake of Cu-ATSM even at constant pO_2_, implicating factors such as variable transporter expression, microenvironmental pH, cellular metabolism or the existence of alternative retention mechanisms [32,45]. Advantages of Cu-ATSM include, rapid uptake, strong signal to noise ratio, the availability of a variety of Cu isotopes with variable half-lives and emission spectra, and some potential for therapeutic as well as diagnostic utility [46-48]. Cu-ATSM agents have subsequently been used to image multiple tumors [16,32,44,46,49-53] and hypoxic tissues [54,55].

In this study, we demonstrate that most (11 of 12) feline head and neck tumors take up ^64^Cu-ATSM with Tav/M and Tmax/M greater than 1.5 and 4.3, respectively. In studies that have investigated Cu-ATSM in human cancer patients, Tav/M ratios ranging from 2.6 – 3.5 have been used as arbitrary cutoff points for defining hypoxic and normoxic tumors [56]. Indeed these levels of radionuclide uptake have been associated with clinically relevant endpoints such as response to treatment and survival. However, these studies have not documented intratumoral hypoxia using independent methods making it difficult to determine whether these T/M ratios are best for determining actual hypoxic state. Furthermore, tumors with significant radiopharmaceutical uptake also demonstrate regions with quantitatively low pO_2_ (less than 7.5 mmHg) or an affinity for pimonidazole, a hypoxia specific marker that forms adducts when the pO_2_ is less than 10 mmHg. Conversely, the bone cyst that failed to take up ^64^Cu-ATSM, with T/M ratios was normoxic based on peritumoral pO_2_ measurements. These results support the hypothesis that ^64^Cu-ATSM uptake occurs in hypoxic rather than normoxic feline tumors. However, complete spatial correlation between distribution of ^64^Cu-ATSM was only possible in one case in which the animal died following imaging and the entire tumor, an osteosarcoma, was available for sectioning and evaluation. Additionally, no proof of ^64^Cu-ATSM uptake or lack thereof in these tumors’ normoxic cells was available. Subjectively, there appeared to be concordance between pimonidazole and ^64^Cu-ATSM uptake. Interestingly, in a xenograft study, ^64^Cu-ATSM uptake failed to correlate with nitroimidazole staining in a sarcoma, while demonstrating a strong correlation in both a carcinoma and a glioma [32].

While we were able to measure hypoxia using at least one other technique in 11 of the 12 tumors, technical problems precluded the use of all three techniques in every case. The intratumoral probe was not operational at the time of evaluation of the first three cats. We also limited our quantitation of tumor pO_2_ to a small number of regions within the tumor. Studies of human tumors suggest that dozens of measurements may be needed to fully map tumor oxygenation. However, our goal was simply to verify the presence or absence of hypoxia in a few intratumoral or peritumoral areas rather than to provide a complete mapping of each tumor.

While the use of pimonidazole has been studied in the dog [33,34], we were unable to find reports of the use of this marker in cats. Therefore, doses were selected based on those reported in dogs. Many drugs, including the nitroimidazole, metronidazole, have similar or identical doses in both cat and dog. We were unable to perform additional procedures such as biopsy in the imaging facility, which necessitated a separate anesthetic episode. Our initial plan had been to administer the pimonidazole concomitant with the ^64^Cu-ATSM to permit side-by-side comparison between the two. However, at the doses used, we were not able to detect pimonidazole in cat biopsy samples collected 24 hours after administration. In contrast, pimonidazole staining was strong and easily visualized when pimonidazole was administered shortly before biopsy. These data suggest that the pimonidazole adducts may turn over more quickly in feline tumors than in dogs [33]. Factors that may have influenced pimonidazole staining intensity in the cat include species specific pharmacokinetic variables such as serum half life, which in humans is about 5 hours and only 15 minutes in the mouse. Therefore recommended doses are several times higher in the mouse than in humans. Unfortunately, these data are not available for the cat. It is possible that with far larger doses of pimonidazole we would have been able to visualize adducts in our biopsy specimens obtained 24 hours after administration. Other factors that could have contributed to poor retention of pimonidazole in tissues include rapidly changing tissue perfusion or rapid turnover of cells in the tumor. HNSCC in cats is considered a rapidly progressive malignancy therefore it is possible that tumor growth kinetics may have also contributed. Pimonidazole dose optimization should be performed in feline tumors to better utilize this technique.

It is not surprising to see heterogeneous distribution of hypoxia within tumors, therefore significant differences between the T_max_/M and T_av_/M in these PET studies is expected. However, signal voids were also observed in areas with poor perfusion (based on CT contrast studies), which would presumably be hypoxic. In one cat with osteosarcoma, the signal void corresponded to a necrotic cavity identified at necropsy. It is possible that poorly perfused regions contain necrotic rather than viable cells. Since uptake and retention of Cu-ATSM requires intact cell and likely lysosomal membranes, it is unlikely that Cu-ATSM would accumulate in these necrotic regions [45]. A compounding factor in the specific case of the osteosarcoma may be the high interstitial pressures in bony areas of osteosarcomas leading to decreased perfusion [57-59].

In this study, while strongest ^64^Cu-ATSM uptake was observed in HNSCC, sarcomas and benign tumors also exhibited uptake and significant hypoxia. Thus, hypoxia is not a characteristic of tumor type or malignancy. The increased uptake among feline HNSCC coupled with intratumoral probe and pimonidazole data support that these tumors are significantly hypoxic like their human counterpart. However, we cannot rule out that some other characteristic of HNSCC, in addition to hypoxia, has influenced Cu-ATSM uptake and retention such as the expression of specific transporters or metabolism. It has been hypothesized that altered redox state associated with glycolytic metabolism in some tumors might also promote reduction and trapping of Cu-ATSM. It is likely that the use of multiple methods to investigate tumor hypoxia may yield the most accurate assessment.

Regardless of whether ^64^Cu-ATSM uptake is a direct reflection of tumor hypoxia, studies of human HNSCC indicate the clinical significance of this tracer. SUV_max_[53] and T_av_/M ratio [56,60] cutoffs have been successfully used to predict recurrence after radiation and prognosis, respectively, in human cancer patients. It was not our objective to correlate these data with prognosis in cats nor was it feasible given inconsistent follow-up therapy in these cases. However, in using spontaneous HNSCC to investigate the biologic impact of therapeutic intervention, these data may guide selection of appropriate thresholds.

Unexpectedly, certain other tissues in these cats exhibited ^64^Cu-ATSM uptake. Uptake in lymph nodes draining the primary tumor was seen in 8/12 cats. These lymph nodes exhibited normal contrast enhancement on CT and only mild to moderate enlargement. In one case, the lymph nodes exhibited reactivity rather than metastasis. While hypoxia is recognized in metastatic or primary tumors occurring in lymph nodes, its presence in reactive lymph nodes has not been previously documented, to the authors’ knowledge [61,62]. It is interesting to consider how hypoxia in draining lymph nodes might influence the development of regional metastasis. Two cats also had ^64^Cu-ATSM uptake in association with presumptive otitis media. Hypoxia has been demonstrated in rats with otitis media [63].

Hyperthyroidism is common in elderly felines and occurs secondary to adenomatous hyperplasia, thyroid adenomas, or least commonly functional thyroid carcinomas [64]. Two of the three patients with ^64^Cu-ATSM uptake in the thyroid had clinically proven functional hyperthyroidism prior to the scan. There are limited data concerning hypoxia in non-malignant disorders of the thyroid, though low level vascular endothelial growth factor (VEGF) expression, which is hypoxia inducible, has been observed in follicular adenomas and adenomatous goiter of the thyroid in humans [65]. This may be caused by the hypermetabolic state and increased oxygen consumption [66] of the thyroid cells in these conditions. Human thyroid carcinoma metastases, though not present in these patients, were also demonstrated hypoxic when imaged with ^99m^Tc-HL91, a nitroimidazole, and SPECT [67]. Confirmation of hypoxia in other tissues using another technique could not be easily performed in these cases due to inaccessibility of lesions and invasive nature of the other techniques used.

Two cats were evaluated before and after different antivascular therapies. It has been proposed that modulation of tumor vasculature may affect intratumoral hypoxia and preclinical studies have supported this notion [1,68]. In this study, treatment was accompanied by only slight changes in Cu-ATSM uptake. Since we do not have data from untreated cats to demonstrate pattern on Cu-ATSM uptake over time, it is not possible to determine whether the changes observed were drug specific. However, in both cats, there was a slight increase in T_max_/M possibly indicating regional vascular compromise. However, these changes may be within range of error, as the inverse quartic relationship between partial pressure of oxygen and Cu-ATSM uptake results in steep slope within the initial decline of pO_2_, while at low pO_2_, slight changes may be insufficient to alter uptake of ^64^Cu-ATSM [13]. At the same time, in the cat treated with a tyrosine kinase inhibitor targeting VEGFR2, a slight decrease in T_av_/M occurred concomitantly with increased quantitative pO_2_ as measured with the intratumoral probe. Furthermore, focal areas in the periphery of the tumors had decreased signal, suggesting that further investigation into dose and time frame of anti-angiogenic therapy administration as a hypoxia modulator might be useful.

Despite their experimental utility, rodent models fail to completely recapitulate human cancer and to provide the degree of heterogeneity that is characteristic of human clinical populations. The gap between xenograft and genetically-engineered mouse models and human clinical studies are well recognized. Furthermore, as function of animal size, the tumors seen are considerably smaller from that expected in a human clinical population. Feline HNSCC may provide a relevant alternative to rodent models for this disease.

## Conclusions

All of the feline HNSCC studied exhibited regional evidence of biologically relevant hypoxia, regardless of measurement technique. Therefore, in addition to morphologic, clinical and molecular similarities, feline and human HNSCC also share physiologic characteristics, further demonstrating how closely the disease in cats mimics its human counterpart. We also preliminarily illustrate, using anti-vascular agents, that feline tumors can be used to study the biologic consequences of interventions and to develop and apply surrogate endpoints. It is reasonable to assume that such studies could be used to address specific issues of clinical translation and inform the development of more effective human trials.

## Abbreviations

HNSCC: Head and neck squamous cell carcinoma; ATSM: Diacetyl-bis(N4-methylthiosemicarbazone); PET/CT: Positron emission tomography/computed tomography; Tmax/M: Ratio of maximum tumor uptake to muscle uptake; Tav/M: Ration of average tumor uptake to muscle uptake; EGFR: Epidermal growth factor receptor; Cox-2: Cyclo-oxygenase isoform 2; MR: Magnetic resonance; SPECT: Single photon emmision computed tomography; FDG: Fluoro-D-Glucose; VEFGR2: Vascular endothelial growth factor receptor 2; SUVbw: Standardized uptake value adjusted for body weight; HU: Hounsfield Unit; RECIST: Response evaluation criteria in solid tumors

## Competing interests

The authors declare that they have no competing interests.

## Authors’ contributions

EAB was responsible for image interpretation and analysis and manuscript preparation. NJM contributed to study design, case recruitment, O_2_ measurements, data management, table and figure preparation. KLB was involved in study design, oversight of imaging, and manuscript editing. DWA was involved in study design, histologic evaluation of biopsies and pimonidazole staining and manuscript review. EAM was responsible for study design, patient recruitment, clinical procedures, imaging, O_2_ measurement, data analysis, and manuscript preparation. All authors read and approved the final manuscript.

## Pre-publication history

The pre-publication history for this paper can be accessed here:

http://www.biomedcentral.com/1471-2407/13/218/prepub
